# Alarmingly High HIV Prevalence Among Adolescent and Young Men Who have Sex with Men (MSM) in Urban Indonesia

**DOI:** 10.1007/s10461-021-03347-0

**Published:** 2021-06-18

**Authors:** Lisa G. Johnston, Phyumar Soe, Asti Setiawati Widihastuti, Artha Camellia, Tarinanda Adzani Putri, Fani Fadillah Rakhmat, Risky Annisa Nurwandani, Shirley Mark Prabhu, Nurjannah Sulaiman, Paul M. Pronyk

**Affiliations:** 1 UNICEF, Bangkok, Thailand; 2 UNAIDS, Yangon, Myanmar; 3 UNICEF Indonesia, Jakarta, Indonesia; 4 University of Padjadjaran, Bandung, Indonesia; 5 UNICEF East Asia and the Pacific Regional Office, Bangkok, Thailand; 6 Ministry of Health, Jakarta, Indonesia

**Keywords:** HIV, Epidemiology, Key populations, Men-who-have-sex-with-men (MSM), Indonesia

## Abstract

Indonesia’s HIV epidemic is concentrated among key populations. While prevalence among men who have sex with men (MSM) is high, transmission among young MSM (15–24-years-old) remains poorly understood. We conducted a respondent driven sampling survey of 211 young MSM in urban Bandung, Indonesia in 2018–2019 to estimate HIV prevalence and associated risk factors. Thirty percent of young MSM were HIV antibody positive. This is nearly 100-fold greater than Indonesia’s population prevalence and sevenfold higher than average estimates for young MSM across Asia and the Pacific Region. Individual risk factors associated with HIV infection were being 20–24 years old, having a steady partner and preferring the receptive position during sex. Issues of stigma, discrimination and social exclusion were common. Few young MSM who were open with friends and family members about their sexual identity. Among those that were, close to half reported experiencing feelings of aversion from these groups. Wider structural factors that reduce social tolerance, restrict the rights of young MSM and compel concealment of sexual identity are likely to fuel high-risk behaviors and limit access to essential testing care and support services including pre-exposure prophylaxis which is not yet widely available. Urgent health, social, legal and political actions are required to respond to these factors and reduce the disproportionate contribution of young MSM to Indonesia’s HIV epidemic.

**Supplementary Information:**

The online version contains supplementary material available at 10.1007/s10461-021-03347-0.

## Introduction

While countries in Asia and the Pacific have witnessed declining HIV infections over the past decade, transmission among key populations remains a serious concern [1]. Men who have sex with men (MSM) account for nearly one-third of new infections in the region, with several countries confronting high prevalence and increasing trends [2].

Young MSM (15–24 years old) are at high-risk of HIV infection [1]. Recent evidence from China and Thailand document higher HIV incidence among young MSM relative to older cohorts [3, 4]. In Malaysia, a 2.5-fold increase in prevalence among young MSM was documented between 2014 and 2017 [1].

Factors contributing to HIV transmission among MSM in the region include the use of social media and entertainment venues (i.e. bath houses) to seek casual sexual partners, high-risk sex associated with alcohol and recreational drug use [5–9] and concurrent sexually transmitted infections [10–12]. While specific vulnerabilities to HIV infection experienced by young MSM are less well understood, lower levels of HIV awareness and self-perceived risk, peer influence from older MSM and the exchange of sex for resources have been identified as transmission drivers [5]. Furthermore, well-documented vulnerabilities to HIV infection experienced by young people globally including limited life skills and agency, sexual coercion and feelings of stigma and social exclusion may contribute to transmission among young MSM in Asia and the Pacific [13].

Indonesia’s HIV epidemic is concentrated among key populations. Apart from two provinces in eastern Indonesia with a low-prevalence generalized epidemic (2.4%), adult HIV prevalence has stabilized at around 0.4%, and new infections decreased by one-third over the past decade [14, 15]. Among key populations, while transmission among female sex workers and people who inject drugs has been stable or declining, HIV prevalence among MSM increased at least three-fold in the past decade—from 5.3% in 2007 to 17.9% in 2019 [15].

Despite concerns of increasing transmission, Indonesia’s HIV epidemic among young MSM remains poorly understood. Data disaggregated from national assessments among young MSM in urban centers found a fourfold increase in HIV prevalence between 2011 and 2015 (from 3.8% to 15.6%) [16]. The most recent national assessment (2019), which found high prevalence among urban MSM (17.9%), has not been disaggregated for young MSM [17]. However, as these estimates are drawn from convenience samples they should be interpreted cautiously.

To better understand young MSM’s contribution to Indonesia’s key population HIV epidemic, we conducted a survey using respondent driven sampling (RDS) among young MSM in Bandung city. We present estimates of HIV prevalence and risk behaviors alongside factors associated with HIV infection and compare these to HIV prevalence estimates among young MSM from countries across the wider Asia and the Pacific region.

## Methods

Data were collected using a cross sectional design over a three-month period between 2018 and 2019 in Bandung, Indonesia’s third most populous urban centre (population 2.4 million). Eligible young MSM were defined as males 15–24 years old who had anal sex with a male in the past six months and lived, worked, or studied in Bandung. This work was embedded within a wider risk assessment of key populations conducted by the University of Padjadjaran (Bandung) and UNICEF Indonesia in partnership with National and Provincial Ministries of Health [18]. The Institutional Review Board at the University of Padjadjaran reviewed and approved the study protocol (08/UN6.C10/PN/2019).

### Respondent Driven Sampling and Survey Procedures

Respondent driven sampling has been widely used to sample socially networked key populations and is described in detail elsewhere [19, 20]. Briefly, RDS is a chain referral sampling method where hard-to-reach populations are asked to refer other members of their social network to enrol in a survey. Respondents provide their social network size (i.e., how many people they know who know them, who fulfil the eligibility and they have seen in the previous two weeks) which is used during analysis to mitigate biases associated with chain referral methods. The sample size was calculated at 300 based on ever having had an HIV test, with 95% confidence, 80% power and a design effect of 2 (a correction factor to increase the sample size to account for the sampling method not being a simple random sample).

Three ‘seeds’ (i.e., initial recruits) were non-randomly selected based on their ability to recruit diverse members of their social networks. Once seeds completed the survey process, they received three uniquely coded coupons to use for recruiting other eligible young MSM. Recruits presenting a coupon at one of two interview sites completed eligibility screening, informed consent and a supervised, self-administered interview with responses entered directly into a tablet. Questionnaires were extensively pre-tested and appropriately revised. Final survey questions recorded socio-demographic details, HIV-related knowledge, beliefs, stigma and discrimination, violence and risk behavior relevant for MSM. Respondents received compensation of approximately 5 USD for survey enrollment and completion and an additional compensation (a maximum of three) for each recruit who enrolled in and completed the survey.

### Laboratory Analysis

All respondents received HIV antibody testing after informed written consent and pre-test counselling. Venous blood samples were collected and analyzed at an accredited national laboratory using standardized protocols. Test results were linked using respondents' unique identification number, a laboratory code and collection date. Respondents received vouchers with identification numbers to use for calling the testing center to receive their test results. Negative antibody results were provided over the phone, while those with positive antibody results were asked to present for further counseling, repeat testing and treatment.

### Measures

The survey instrument was developed using questions from HIV Integrated Behavioural and Biological Surveys (IBBS) conducted in the Asia Pacific region and the Global AIDS indicators 2020 [21]. Some indicators were collapsed due to the small category size. For instance, the question asking participants how they would best describe their sexual identity had responses for “homosexual”, “heterosexual”, “bi-sexual”, “transgender” and “other”. Given that few MSM responded as being “heterosexual” (n = 8), this category was collapsed with “bisexual”. Comprehensive HIV knowledge was measured by having correct answers about whether “HIV transmission can be reduced by having sex with only one uninfected partner who has no other partners”, “a person can reduce their risk of HIV infection by using a condom every time they have sex”, “a healthy-looking person can still have HIV”, “a person can become HIV infected from mosquito bites” and “a person can get HIV by sharing food with someone who is infected” [21]. A paying partner was described to participants as someone from whom they sold anal sex in exchange for money or goods. A steady partner was described to participants as a husband or wife, boyfriend or girlfriend or someone considered to be a permanent partner. A non-regular, casual partner was described as someone whom they did not consider to be a steady partner, with whom they had sexual intercourse occasionally or once. Participants were asked to assess their own risk for HIV infection to which they could respond “high risk”, “some risk”, “low risk”, “already living with HIV” or “do not know”. “High risk” and “some-risk” were collapsed together and living with HIV or do not know were removed from the denominator.

To contextualize our findings, estimates of HIV prevalence for MSM were compiled for countries in the Asia and the Pacific region from the UNAIDS Key Population Atlas [22]. HIV prevalence estimates are reported by countries to UNAIDS based on findings from bio-behavioral surveillance surveys and may reflect sub-national estimates, rather than national estimates.

### Statistical Analysis

All variables were assessed for biases related to RDS assumptions using convergence and bottleneck plots and homophily values [23] in RDS Analyst (www.hpmrg.org), an open-source software based in R program with a graphical user interface specific to RDS analysis [21]. All analyses were conducted using the Gile successive sampling estimator in RDS Analyst using the following cascade of questions to measure network size: (1) “How many males do you know and they know you who are between the ages of 15–24 years who had anal sex with a male in the past six months?”, (2) How many of them live, work, or study in Bandung?”, and (3) How many of them have you seen in the last 2 weeks? The response to the third question was used as the participant’s social network size. Bivariate regression to assess associations between socio-demographic and behavioral variables with HIV status was conducted in STATA v.13 using exported successive sampling weights.

Variables associated with HIV status (p < 0.2) in bivariate analysis or considered important confounders were included in the multivariable model. Adjusted odds ratios (aOR) and 95% confidence intervals (CI) are presented in the final model. Missing values were omitted from the analyses.

## Results

### Characteristics of Young MSM

Over the course of 34 days, 211 young MSM were sampled. The final sample had three seeds and a maximum number of ten recruitment waves. All variables reached convergence in advance of the final estimates, had neither high homophily nor heterophily and resulted in no bottlenecks. Baseline characteristics are presented in Table 1. Most young MSM are between the ages of 20–24 years old and were employed. Equal proportions of respondents (half) reported having a steady and/or casual partners. Two-thirds used a condom during their last anal sex with steady, casual and paying partners. Roughly one-third have comprehensive knowledge of HIV and report knowing that their steady or casual partners have other partners. One third received money for sex. Equal percentages (one-third) preferred receptive intercourse, insertive intercourse or had no preference for either position. Among half of young MSM reporting alcohol use, two-thirds had sexual intercourse while intoxicated. Of the few that use drugs, the majority used sedatives or cannabis. Just 13% have disclosed that they had sex with men to their families, among which one-third experience stigma and discrimination from friends or family. One fifth experienced sexually transmitted infections symptoms in the past year. Thirty percent tested HIV antibody positive. Among all young MSM, 13% knew their HIV status in advance and 17% learned their HIV status during the survey.

**Table 1:** Characteristics of young MSM (total and by HIV status) in Bandung, Indonesia

	Total young MSM = 211			HIV negative young MSM = 146			HIV positive Young MSM = 63		
	n/N	%	% (95% CI)	n/N	%	95% CI	n/N	%	95% CI
Socio-demographic characteristics									
Median age (mean, median, range)	20.6, 21 (15–24)			20, 22 (15–24)			22, 23(17–24)		
Age group									
15–19	75/209	32.4	25.3, 40.4	68	41.7	32.3, 51.7	6	11.0	3.9, 27.1
20–24	134/209	67.6	59.6, 74.7	76	58.3	48.3, 67.7	57	89.0	72.9, 96.1
Currently enrolled in school	77/205	37.4	30, 44.9	60	39.9	30.4, 50.2	17	32.8	19.4, 49.7
Currently employed	143/209	64.8	56.0, 72.7	93	62.8	52.3, 72.3	48	68.4	51.3, 81.6
Sexual identity									
Homosexual	115/211	56.5	48.1, 64.6	76	54.7	44.5, 64.6	38	61.8	46.6, 75.0
Bisexual	87/211	38.5	30.7, 46.8	61	38.4	29.2, 48.6	25	38.2	25.0, 53.4
Other	9/211	5	2.3, 10.7	7	6.9	3.0, 15.0	0		
Alcohol and substance use									
Alcohol past 6 months	105/211	48.7	40, 57.5	77	51.2	41.0, 61.4	26	41.5	27.3, 57.3
Drug use (ever)	29/209	15.4	10.3, 22.5	22	16.6	10.5, 25.2	5	10.7	3.7, 27.0
Injectable drugs among those who have used drugs	2/29	5.3	1.1, 21.5	0			1	9.7	0.01, 55.3
Knowledge and perceptions									
Ever received information on HIV prevention	154/202	78.8	71.3, 84.8	98	72.8	64.4, 81.1	54	91.8	86.2, 97.6
Self-perceived HIV risk (high or some)	115/194	64	55.5, 71.8	79	59.0	48.9, 68.5	35	78.1	63.3, 88.1
Comprehensive knowledge of HIV	63/211	33.7	25.9, 42.6	29	23.2	15.2, 33.9	34	59.2	44.0, 72.7
HIV risk behaviour									
Age of first anal sex (mean, median, range)	18, 18 (8–24)			17.8, 18 (8–24)			18.3, 18 (12–22)		
Type of sex partner (past year)									
Non-paying, steady partner	112/209	52.5	44.4, 60.9	59	37.9	28.6, 48.3	52	85.8	74.4, 92.7
Non paying, non-regular, casual partner	97/209	49.2	40.7, 57.7	64	46.5	36.5, 56.8	31	54.1	38.8, 68.6
Transgender	48/209	24.3	17.9, 32.1	45	32.4	23.8, 42.4	3	6.0	1.8, 18.3
Exchange of sex for resources									
Paid for sex	34/209	16.9	11.4, 24.5	28	19.9	12.9, 29.2	5	10.2	3.4, 26.7
Received payment for sex	74/203	31.4	24.2, 39.6	52	32.2	23.7, 42.1	20	27.8	15.8, 44.0
On-line/social media client identification^b^	43/74	61	46.2, 73.6	26	47.3	31.5, 63.7	15	92.4	74.1, 97.5
Preferred sexual position									
Insertive	74/209	35	27.4, 43.5	59	40.7	31.1, 51.1	14	22.1	11.8, 37.5
Receptive	76/209	33.7	26.2, 42.1	46	28.5	20.4, 38.4	30	46.5	31.8, 61.8
No preference	59/209	31.3	23.8, 39.7	39	30.8	21.9, 41.3	19	31.4	19.2, 46.9
Sexual violence									
First sexual experience forced	60/185	35.7	27.5, 44.9	41	36.5	26.4, 47.9	17	32.6	19.6, 49.0
Used condom at last sex^a^									
Steady partner	71/111	66.2	54.3, 76.4	35	60.2	43.1, 75.1	35	71.7	54.3, 84.3
Casual partner	66/97	67.4	54.7, 78.0	41	62.1	45.8, 76.1	23	76.0	56.1, 88.7
Paying partner	47/72	66.9	53.0, 78.4	28	60.7	44.0, 75.1	17	79.5	51.4, 93.4
Transgender partner	23/48	49.5	35.3, 64.2	20	45.6	29.0, 63.2	3	100.0	
Partner concurrence^a^									
Steady partner has other partners	16/71	29.5	17.1, 42.6	11	33.5	18.6, 52.7	5	27.3	10.6, 54.3
Casual partner has other partners	36/95	37.9	27.1, 48.7	25	44.4	29.6, 60.3	10	25.2	12.3, 44.8
Sex while under the influence of alcohol	70/211	34.5	26.9, 42.8	53	37.6	28.4, 47.8	15	26.0	14.3, 42.5
Sexually transmitted infections									
STI symptoms in past 12 months	47/209	21.4	15.6,28.6	29	20.8	13.8, 30.1	17	21.6	12.8, 34.2
Stigma and discrimination									
Family knows participant has sex with males	29/209	13.1	8.3, 19.7	14	11.1	5.8, 20.2	15	17.7	9.9, 29.8
Experience of aversion from family/friends	11/29	41.9	20.4, 67.0	4	46.3	16.6, 78.8	7	35.7	14.2, 65.1
Experience of aversion from health staff	9/209	2.9	1.4, 5.9	5	2.3	1.7, 4.4	3	3.6	0, 7.7
Access to HIV prevention services									
Received counselling on condom use/safe sex in past 3 months	86/195	42.5	34.1, 51.4	53	38.5	28.7, 49.4	31	50.1	34.8, 65.4
Received HIV test and test results in the last 12 months	63/211	28.6	21.7,36.6	35	23.8	16.1, 33.6	26	38.1	24.9, 53.4
Tested for sexually transmitted infections in past three months	48/196	22.1	16.0, 29.8	27	16.5	10.5, 25.0	19	34.2	21.0, 50.5
HIV PREVALENCE									
Total	63/211	30.3	22.2, 38.4						

^a^Among those who had respective partners in the last 12 months

^b^AMONG those who sold anal sex for money or goods in past year

### Associations with HIV Positivity

The risk factor analysis is presented in Table 2. HIV antibody positive young MSM have higher odds of being older (20–24 years old versus 15–19 years old), having received information on HIV, having higher comprehensive knowledge, perceiving themselves to have some or high risk of infection, having a steady partner, using on-line resources to identify clients to exchange sex for resources, preferring the receptive position during anal sex, having test for an sexually transmitted infections in the past three months and lower odds of having had sex with a transgender person in the previous year.

**Table 2:** Bivariate and multivariate analysis of the association between study variables and HIV infection among young MSM, Bandung, Indonesia

	Bivariate		Multivariate	
	OR	95% CI	Adjusted OR	95% CI
Socio-demographic characteristics				
Median age (mean, median, range)	1.5***	1.22, 1.86	1.38**	1.12, 1.70
Age group				
15–19	Ref			
20–24	5.82**	1.79, 18.89		
Currently enrolled in school	0.73	0.32, 1.67		
Currently employed	1.28	0.55, 2.96		
Knowledge and perceptions				
Ever received information on HIV prevention	4.27**	1.60, 11.41		
Self-perceived HIV risk (high or some)	2.45*	1.06, 5.67		
Comprehensive knowledge of HIV	4.79***	2.14, 10.72		
HIV risk behaviour				
Type of sex partner (past year)				
Non-paying, steady partner	9.92***	4.27, 23.06	6.01***	2.37,15.20
Transgender	0.13**	0.04, 0.50		
Preferred sexual position				
Insertive	Ref		Ref	
Receptive	3.01*	1.17, 7.71	3.02*	1.10, 8.33
No preference	1.88	0.70, 5.06	1.82	0.56, 5.91
Access to hiv prevention services				
Tested for sexually transmitted infections in past three months	2.63*	1.12, 6.19		

^*^p < 0.05

^**^p < 0.01

^***^p < 0.001

In the multivariable model, being 20–24 years old, having a steady partner and preference for the receptive position are associated with HIV positivity. No differences were observed in a sub-analysis comparing young MSM who had prior knowledge of their HIV antibody positive status to those who did not (data not shown).

### Asia and the Pacific Country Comparison

**Fig. 1:**
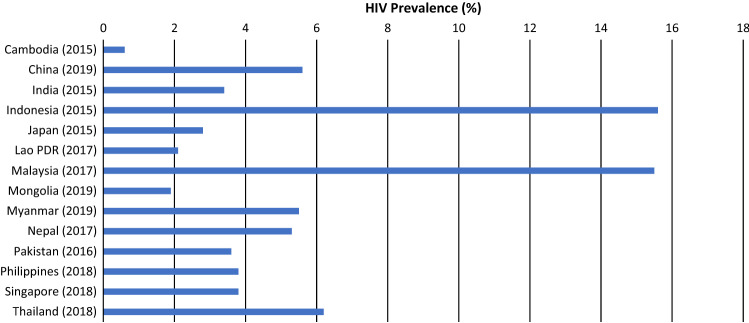
HIV prevalence among young MSM (15–24 years old) in Asia and the Pacific Region*

To contextualize our findings, we compiled estimates of HIV prevalence among MSM from 26 countries in Asia and the Pacific (web appendix). Figure 1 presents data reported by 18 countries in the region where recent national estimates of HIV prevalence among young MSM are available (2015 onwards). HIV prevalence averaged (unweighted) 6.2% for all MSM, and 3.9% for young MSM. Prevalence among MSM aged 25 years or older was 2.2-fold higher than for young MSM.

## Discussion

Nearly one third of young MSM in Bandung city are living with HIV, which is among the highest prevalence reported globally for this age group. HIV prevalence among young MSM in Bandung is nearly two-fold higher than reported prevalence for MSM of all ages from a national assessment conducted during approximately the same period, and 100-fold higher than national prevalence estimates among the general population. With stable or declining prevalence in other key population groups, high HIV prevalence among young MSM reflects recent infections which likely contribute disproportionately to the country’s HIV epidemic [15]. Indonesia’s escalating HIV infections among young MSM take place on a backdrop of relatively flat trends for MSM across Asia and the Pacific, with prevalence estimates observed in this study seven-fold higher than regional averages from countries where recent data are available.

Few socio-demographic or behavioral factors explain differences between HIV antibody positive and negative young MSM. Young MSM older than 20 years, those with a steady partner and who prefer the receptive position during sex were at higher risk of HIV infection. Findings regarding higher levels of knowledge, self-perceived risk and testing for sexually transmitted infections which were significant in the unadjusted analysis only are likely mediated by the effects of age and/or HIV status. Our findings related to low comprehensive HIV knowledge, low drug and alcohol use, high perception of infection risk and high levels of condom use among young MSM are largely consistent with recent national estimates for MSM of all ages [15].

Partner concurrency and sexually transmitted infections may play an important role in transmission. While not associated with HIV antibody positivity in our study, one-third of young MSM reported their steady or casual partners had other partners. Our findings that one-fifth of young MSM experienced sexually transmitted infections symptoms in the past 6 months align with recent biological estimates of high rates of gonorrhea (18.7%), chlamydia (27.9%) and syphilis (9.6%) among MSM country-wide [15]. Two thirds of young MSM reported condom use at last sex with steady or casual partners.

The relative absence of individual-level associations points to the role of wider structural factors in shaping the risk environment for young MSM [24]. Among the just 13 percent of young MSM were open with friends and family members about their sexual identity, close to half reported experiencing aversion from these groups. Recent qualitative research in Indonesia highlights pervasive bullying of sexual and gender minority youth in school settings [25], with concealment of sexual identity among MSM being an important contributor to high-risk behavior [26, 27]. These findings are re-enforced by an assessment of levels of acceptance of homosexuality among 34 countries, where Indonesia is ranked consistently near the bottom [28]. Persistent calls for criminalization of homosexuality and police crackdowns on MSM are a serious concern [29].

Structural barriers also limit access to critical testing and support services. Despite high perceived HIV risk, less than one third of young MSM had an HIV test in the past year which is half the national levels for all MSM [15]. This may be partly due to legal requirements for parent or guardian consent for those under 18 years old and conflicting laws and regulations that are punitive towards MSM including the criminalization of HIV transmission, exposure, and nondisclosure of HIV status [30]. Fortunately, young MSM interacting with the health sector report experiencing little stigma and discrimination. Among young MSM aware of their HIV antibody positive status in our study almost all had initiated anti-retroviral therapy. Pre-exposure prophylaxis (PrEP) has not yet been widely implemented in Indonesia.

While this study is among the first RDS assessments of young MSM in Indonesia, there are several limitations. First, respondents in Bandung do not represent young MSM in all urban centers. However, among six major cites included in Indonesia’s 2015 national assessment, HIV prevalence among MSM (all ages) in Bandung was in the middle range at 28% (range 13.2–36.9%). Comparisons with other existing data from the region should be interpreted with some caution as they were collected during different time periods and many used non comparable sampling methods, including convenience sampling. Second, the projected sample size of 300 respondents was not reached, potentially because it was too large relative to the young MSM population. Nonetheless, population saturation appears to have been reached near survey completion. Although young MSM were given a number to call to get their HIV test results, many did not. This is especially concerning since many were found to be living with HIV still be unaware.

## Conclusion

In summary, urgent health, social and legal measures are required to improve access to essential services and address the structural drivers of accelerating HIV transmission among young MSM in urban Indonesia. These include improving national and sub-national coordination to ensure counselling, testing and treatment are widely available including the removal of age-related restrictions; sexually transmitted infections screening and management; accelerating efforts to pilot and scale PrEP; mobilizing peer outreach and referrals; optimizing the use of social media; enhancing cross-ministerial efforts between health and education to strengthen school-based primary prevention efforts to improve sexual health awareness; revising legal frameworks to reduce stigma and discrimination; and responding to the mental health and psycho-social needs of young MSM.

## Supplementary Information

Below is the link to the electronic supplementary material.

Supplementary file1 (XLSX 15 kb)

Supplementary file2 (DOCX 15 kb)
