# Supplementary material for: Alarmingly High HIV Prevalence Among Adolescent and Young Men Who have Sex with Men (MSM) in Urban Indonesia
Source: AIDS Behav. 2021 Jun 18;25(11):3687–94. doi: 10.1007/s10461-021-03347-0 (PMC8560664; doi:10.1007/s10461-021-03347-0)
Supplement: Supplementary file 2 — Supplementary file2 (DOCX 15 kb) [file 10461_2021_3347_MOESM2_ESM.docx]

**WEB APPENDIX: HIV prevalence among MSM in countries in Asia and the Pacific Region: By age-group and change over time**

The most recent HIV prevalence estimates for all MSM averaged 6.2% (range: 0.2% Bangladesh - 21.6 % Malaysia). Among 18 countries with at least two national assessments in the past decade, the average annual increase in HIV prevalence was 0.38%. HIV prevalence among MSM declined in one country, was stable in ten countries (annual HIV prevalence change neutral +/- 0.2%) and increased in seven countries.
